# Factors of Muscle Quality and Determinants of Muscle Strength: A Systematic Literature Review

**DOI:** 10.3390/healthcare10101937

**Published:** 2022-10-03

**Authors:** Luciano Bruno Kuschel, Dominik Sonnenburg, Tilman Engel

**Affiliations:** Department of Sports Medicine and Sports Orthopedics, University Outpatient Clinic, University of Potsdam, 14469 Potsdam, Germany

**Keywords:** muscle quality, muscle strength, phase angle, echo intensity

## Abstract

Muscle quality defined as the ratio of muscle strength to muscle mass disregards underlying factors which influence muscle strength. The aim of this review was to investigate the relationship of phase angle (PhA), echo intensity (EI), muscular adipose tissue (MAT), muscle fiber type, fascicle pennation angle (θf), fascicle length (lf), muscle oxidative capacity, insulin sensitivity (IS), neuromuscular activation, and motor unit to muscle strength. PubMed search was performed in 2021. The inclusion criteria were: (i) original research, (ii) human participants, (iii) adults (≥18 years). Exclusion criteria were: (i) no full-text, (ii) non-English or -German language, (iii) pathologies. Forty-one studies were identified. Nine studies found a weak–moderate negative (range r: [−0.26]–[−0.656], *p* < 0.05) correlation between muscle strength and EI. Four studies found a weak–moderate positive correlation (range r: 0.177–0.696, *p* < 0.05) between muscle strength and PhA. Two studies found a moderate-strong negative correlation (range r: [−0.446]–[−0.87], *p* < 0.05) between muscle strength and MAT. Two studies found a weak-strong positive correlation (range r: 0.28–0.907, *p* < 0.05) between θf and muscle strength. Muscle oxidative capacity was found to be a predictor of muscle strength. This review highlights that the current definition of muscle quality should be expanded upon as to encompass all possible factors of muscle quality.

## 1. Introduction

Muscle quality was introduced to help explain age-related and pathological declines in relative muscle strength [1,2,3]. Accordingly, muscle quality has been defined as the ratio of muscle strength to appendicular skeletal muscle mass [1,2] or muscle volume [4]. While describing the term *muscle quality* as relative muscle strength (in relation to muscle mass) might be a good first approach to estimate the strength production capacity of skeletal muscle tissue [5], solely using this definition does not address underlying changes in the skeletal muscle tissue itself. Muscle quality could be an invaluable parameter for assessing physiological properties of the skeletal muscle tissue which influence the strength and the function of the muscle. In addition, changes of muscle quality could help better explain age- and pathology-related losses of muscle strength and muscle function once the field of muscle quality is better understood in the healthy population. However, questions remain as to which underlying factors can be contributed to the term *muscle quality* and how these factors relate individually to muscle strength. In a paper by McGregor et al. [6] the authors discuss potential factors which may underpin muscle quality. These factors were categorized in muscle size, muscle fiber type, muscle architecture, muscle aerobic capacity, intermuscular adipose tissue, muscle fibrosis, and neuromuscular activation [6]. While the review proposes and rationalizes potential indicators of muscle quality [6], it does not answer how these factors relate to muscle strength. Thus, the aim of this systematic literature review was to investigate the relationship between selected muscle-associated physiological factors and muscle strength in healthy adults, in light of a comprehensive definition of muscle quality.

## 2. Materials and Methods

### 2.1. Search Strategy

The online database MEDLINE was systematically searched using the PubMed search engine in October 2021. The search terms were selected based upon synonyms and variations of this study’s predefined factors related to muscle quality and muscle strength, as shown in Table 1. In addition, following PubMed filter settings were selected: (1) PUBLICATION DATE: “10 years”, (2) LANGUAGE: “English and German”, (3) SPECIES: “Humans”, (4) AGE: “Adult: 19+ years”.

### 2.2. Inclusion and Exclusion Criteria

Inclusion criteria were defined as: (i) original research, (ii) human participants, (iii) participants aged 18 years or older, (iv) outcomes related to muscle strength, (v) proposed factors underlying muscle quality (i.e., muscle fiber, muscle satellite cells, muscle fiber contractile properties, muscle fascicle length, pennation angle, phase angle, muscle aerobic capacity, insulin resistance, intermuscular adipose tissue, motor unit number and neuromuscular activation). Exclusion criteria were: (i) no full text available, (ii) other languages than English or German, (iii) participants with pathologies.

### 2.3. Assessment of Study Quality

The methodological quality of the individual articles included within this review were assessed using the National Heart, Lung, and Blood Institute’s (NIH) Study Quality Assessment Tools [7], which is recommended for the evaluation of the methodological quality or the “risk of bias” of studies in the medical field [8]. Each study was rated as *good*, *fair*, or *poor*. These ratings reflected the individual study’s risk of bias. The rating *good* indicated a low risk of bias, whereas *fair* indicated a moderate risk of bias and *poor* reflected a significant risk of bias [7].

## 3. Results

**Figure 1:**
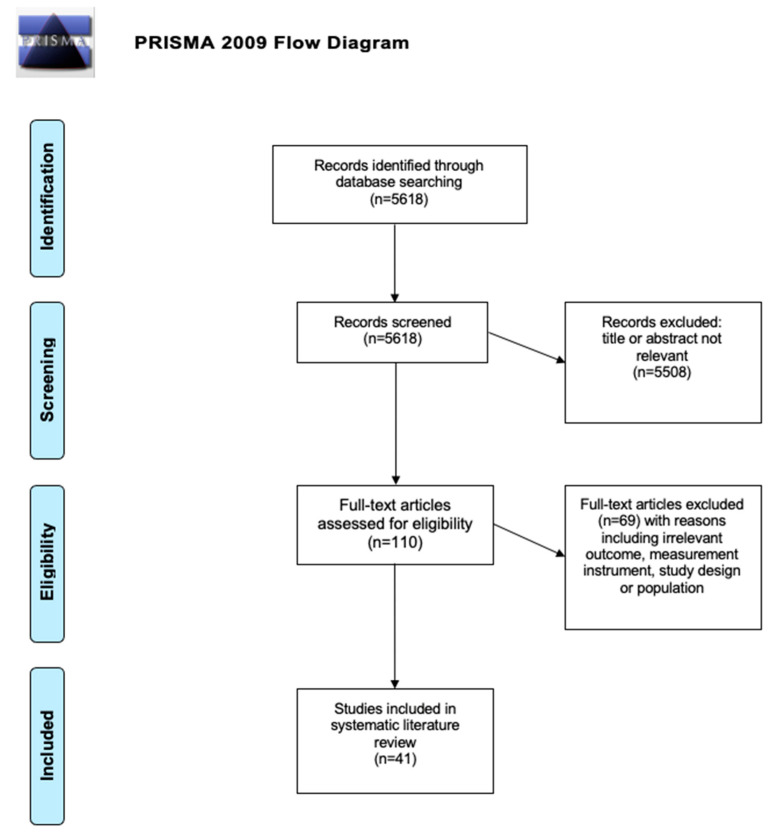
PRISMA Flow Diagram. Summary of the Search Strategy and Selection Process.

A total of 5618 studies were identified during the search process using PubMed. After the screening process, 41 studies were included in the systematic review. Figure 1 presents a flowchart of the literature search according to the PRISMA guidelines. Table 2 shows all included studies with the studies’ inclusion and exclusion criteria, the most relevant findings, the investigated outcomes, and the methodological study quality. All 41 studies were cross-sectional studies by design. The sample of the included studies consisted out of a total of 4449 healthy participants. Almost 70% of the 41 studies assessed muscle strength via isometric or concentric knee extension maximum voluntary contraction (MVC) assessed by dynamometer. The second most used outcome instrument was the hand-held dynamometer which was used to assess the hand grip MVC. In the methodological quality assessment of the 41 included studies, almost half of all the studies received a rating of *fair*, a quarter received a rating of *good* and the remaining quarter received a rating of *poor*. Of particular note was that none of the studies performed a power analysis for sample size calculation.

### 3.1. Muscle Composition

#### 3.1.1. Echo Intensity

Nine studies found a weak to moderate negative correlation (range r: [−0.26]–[−0.656], *p* < 0.05) between muscle strength and EI [9,11,12,17,18,19,20,21,22]. A moderate positive correlation was found between EI and the percent decrease in plantar flexion MVC when comparing the MVC produced at a slower to a faster velocity in the young, old, and combined group (range r: 0.479–0.605, *p* < 0.05) [15]. Other studies found significant correlations between certain parameters of muscle strength and EI in some subgroups or muscles but not between other parameters of muscle strength and EI in other subgroups or muscles [10,14,16,23]. When looking at how EI affected muscle strength, studies found that EI was a predictor of muscle strength (range unstandardized β: [−0.203]–[−0.73], range R^2^: 0.17–0.29, *p* < 0.05) [13,17,20,22]. Inversely, muscle strength was also observed to be a predictor of EI (range R^2^: 0.206–0.3, β: −0.32, *p* < 0.05) [11,12].

#### 3.1.2. Phase Angle

Bittencourt et al. [26] found a weak positive correlation between PhA and muscle strength (r = 0.177, *p* = 0.029) and three other studies found a moderate positive correlation between PhA and muscle strength (range r: 0.422–0.696, *p* < 0.05) [25,27,30]. Contrary, Hetherington-Rauth et al. [28] found no correlation between PhA and HGS in the adult and older adult groups. PhA was found to be a predictor of muscle strength (range R^2^: 0.275–0.71; range unstandardized β: 0.095–24.209, *p* < 0.05) [24,25,29].

#### 3.1.3. Muscular Adipose Tissue

Two studies found a moderate to strong negative correlation between MAT and muscle strength (range r: [−0.446]–[−0.87], *p* < 0.05) [33,34]. Young et al. [31] found a moderate negative correlation between biceps femoris intramuscular adipose tissue (IntraMAT) and both absolute (r = −0.4, *p* < 0.01), and normalized peak leg flexion torque (r = −0.5, *p* < 0.01), and between rectus femoris IntraMAT and normalized leg extension torque (r = −0.4, *p* = 0.01). However, no correlation was found between rectus femoris IntraMAT and absolute leg extension peak torque. Wroblewski et al. [32] also found no correlation between muscle strength and IntraMAT. Inhuber et al. [34] found that MAT was a predictor bilaterally of both knee flexion and extension muscle strength (range *p*: 0.001–0.049).

### 3.2. Muscle Architecture

#### 3.2.1. Fascicle Pennation Angle

Two studies found a weak to strong positive correlation between θf and muscle strength (range r: 0.28–0.907, *p* < 0.05) [44,48]. Other studies found a significant weak to moderate correlation between θf and muscle strength in certain subgroups or muscles but not in other subgroups or muscles [23,43,45,47]. In addition, vastus intermedius muscle θf was found to have a positive influence on isometric knee extension (*p* < 0.01) in young adults but not in older adults [23]. In a study by Trezise et al. [44] θf of the middle vastus lateralis muscle was found to be a predictor of isometric knee extension (R^2^ = 0.72), while θf of the proximal vastus lateralis was a predictor of concentric knee extension (R^2^ = 0.65). Cuesta-Vargas & Gonzalez-Sanchez [46] found the θf of the left-side of the erector spinae to be a predictor of isometric trunk extension (R^2^ = 0.680, standardized β = 0.443, *p* = 0.025). Right side θf of the erector spinae was not found to be a significant predictor of isometric trunk extension MVC [46].

#### 3.2.2. Fascicle Length

Trezise et al. [44] found a weak to moderate positive correlation between isometric knee extension MVC and proximal vastus lateralis muscle lf (r = 0.46, *p* ≤ 0.01), and rectus femoris muscle lf (r = 0.31, *p* ≤ 0.05). A weak positive correlation was also found between concentric knee extension MVC and proximal vastus lateralis muscle lf (r = 0.37, *p* ≤ 0.01), and vastus intermedius muscle lf (r = 0.31, *p* ≤ 0.05). No other significant correlations were observed, and lf was not included in the best fit models for predicting knee extension MVC. Selva Raj et al. [43] found no correlation between neither isometric nor concentric knee extension and vastus lateralis lf. Ando et al. [45] also found no correlation between isometric knee extension and lf of the quadriceps femoris muscles.

#### 3.2.3. Muscle Fiber Type

Herda et al. [40] found a moderate negative correlation between type I percentage of myosin heavy chain (MHC) isoform and isometric knee extension MVC (r = −0.54, *p* = 0.048). No other significant correlations were found when investigating the correlation between types I, IIA and IIX MHC isoforms and isometric and concentric knee extension MVC [40]. Evangelidis et al. [41] found no correlation between the MHC isoform composition of the biceps femoris muscle and isometric knee flexion MVC. De Souza et al. [42] found that the percentage of type II muscle fibers in the vastus lateralis muscle was not significantly different between the high strength performance group and low strength performance group. However, the authors did find that the percentage of type II muscle fibers and the total quadriceps muscle cross-sectional area were predictors of one-repetition maximum (1RM) in the low strength performance group (adjusted R^2^: 0.25, *p* < 0.01) and when both groups were grouped together (adjusted R^2^: 0.35, *p* < 0.01) [42].

### 3.3. Muscle Oxidative Capacity

Zane et al. [49] measured muscle mitochondrial oxidative capacity as the postexercise phosphocreatine resynthesis rate (kPCr) assessed via phosphorus magnetic resonance spectroscopy. The median kPCr was higher (*p* = 0.036) and the percent phosphocreatine (%PCr) depletion was greater (*p* < 0.01) in the higher muscle strength tertiles. kPCr was found to be a predictor of quadriceps muscle strength (β = 0.114, *p* < 0.01) when adjusted for age, sex, height, weight, and %PCr depletion (adjusted R^2^ = 0.531).

### 3.4. Insulin Sensitivity

When comparing the absolute muscle strength of elbow and knee extension and flexion, and hand grip force of two groups with different insulin sensitivities, Gysel et al. [35] found that the less insulin sensitive group and the more sensitive group had similar absolute muscle strength values for all strength outcomes, aside from elbow extension, which was higher in the less insulin sensitive group (+8%, *p* < 0.05). However, when the strength outcomes were normalized to lean mass (i.e., the ratio of torque or force to lean mass) all strength outcomes, aside from elbow extension, were significantly higher in the more insulin sensitive group (*p* < 0.05). Justice et al. [36] found a weak positive correlation between IS and 1RM (r = 0.30, *p* < 0.05), but no correlation between IS and lower body strength. Another study by Gysel et al. [39] found a weak negative correlation between IS and HGS normalized to muscle mass (r = −0.23, *p* < 0.01), but this correlation was not found between IS and non-normalized HGS. Two studies found no correlation between IS and muscle strength [37,38].

### 3.5. Neuromuscular Components

Herda et al. [40] found that the slopes from the MUAPAMPS-RT relationships were significantly correlated with isometric knee extension MVC (r = 0.81, *p* < 0.01) and concentric knee extension MVC (r = 0.79, *p* < 0.01) [40]. Kaya et al. [50] found no main effect of either motor unit size index, nor motor unit number index on isometric pinch grip strength. Trezise et al. [44] found a weak to moderate positive correlation between the electromyography (EMG) amplitude of the knee extensors normalized to their respective M-wave amplitudes (EMG:M) during isometric knee extension MVC (range r: 0.35–0.47, *p* ≤ 0.01), and concentric knee extension MVC (range r: 0.25–0.30, *p* ≤ 0.05). Percent voluntary contraction (%VA) was also found to be significantly correlated with isometric knee MVC (r = 0.25, *p* ≤ 0.05) and concentric knee extension MVC (r = 0.27, *p* ≤ 0.05). The normalized muscle activation of the knee extensors and %VA were also predictors of isometric knee extension MVC (EMG:M: range adjusted R^2^: 0.12–0.22; %VA: adjusted R^2^ = 0.07), and concentric extension MVC (EMG:M: range adjusted R2: 0.06–0.09; %VA: adjusted R^2^ = 0.07). The normalized muscle activation of the whole quadriceps femoris muscle was included in the authors model for predicting isometric knee extension, alongside muscle size and lf (R^2^ = 0.72). However, normalized rectus femoris muscle activation did not correlate with the concentric knee extension MVC, nor was it a predictor of concentric knee extension MVC.

## 4. Discussion

The aim of this systematic review was to establish how the proposed muscle quality factors EI, PhA, MAT, IS, muscle oxidative capacity, lf, θf, muscle fiber type composition, and neuromuscular activation relate to muscle strength. A total of 41 studies met the inclusion criteria for the review. The results indicate that muscle composition assessed via PhA and EI, is associated with muscle strength. MAT, θf, and muscle oxidative capacity are likely also associated with muscle strength but studies that assessed these outcome parameters were scarce in quantity or had a significant risk of bias. No clear association was evident between the factors lf, muscle fiber type, neuromuscular activation, motor unit, IS and muscle strength.

### 4.1. Muscule Composition

#### 4.1.1. Echo Intensity

Nine out of fifteen studies that directly investigated the correlation between EI and muscle strength found a significant negative correlation. The remaining six studies found mixed results, but nonetheless significant correlations were found between some muscle strength measurements and EI measurements. For example, Stock et al. [14] found a moderate negative correlation between isometric knee extension MVC normalized to body mass and subcutaneous fat corrected EI (r = −0.5, *p* < 0.05). However, no correlation was found between normalized isometric knee extension MVC and non-subcutaneous fat corrected EI. Interestingly, other included studies did find non-subcutaneous fat corrected EI to be correlated with muscle strength [18,22]. Such mixed results might be due to differences in utilized US brands, settings, and equations [14,51,52]. Different US brands or systems have been shown to lead to different absolute EI measurements [14,51,52]. While the settings of a US system can be adjusted as to improve EI measurements, these settings are system-specific and cannot be implemented for other US systems [14,51,52]. This issue especially affects EI measurements which use various equations in an attempt to adjust for factors such as adipose tissue [14,51,52], which again, are system-specific and cannot be used for other US systems [14,51]. Considering that the included studies used various US systems to evaluate EI, it seems plausible that some studies would be subject to these systematic errors. In addition, previous studies have shown that the reliability of determining EI is considerably influenced by factors such as adipose tissue, gender, and specific muscle region [51,53,54]. When considering that the samples of the studies had varying BMIs ranging from healthy to overweight, it is possible that adipose tissue may have been partially responsible for the influencing EI measurement which might have been responsible for the mixed results.

EI is commonly used as a measure of skeletal muscle composition due to its ability to assess changes within the muscle, such as intramuscular adipose tissue or intramuscular fibrosus [55,56]. Thus, an increase in EI would indicate an increased amount of MAT and connective tissue and a decrease in contractile tissue [57,58,59,60]. Changes in muscle composition such as an accumulation of MAT have been shown to potentially have a negative effect on muscle strength [61,62,63]. Thus, the relationship between EI and muscle strength might be reflective of changes within the muscle tissue itself which affect the muscle’s force production abilities. According to these results, EI is closely associated with muscle strength.

#### 4.1.2. Phase Angle

All but one study found an association between PhA and muscle strength. Hetherington-Rauth et al. [28] found no correlation between PhA and HGS in the adult and older adult groups. However, other included studies did find an association between PhA and HGS [25,26,27,29,30]. This was also true when comparing studies with similarly aged groups [25,29]. It should be noted that Hetherington-Rauth et al. [28] did find a significant correlation between PhA and HG MVC in the youth group. PhA is a measure of capacitive and resistive properties of bodily cells [64], and as such the connection between PhA and muscle strength is likely due to the association of PhA to lean body mass [65], and intra- and extracellular fluid [64]. While the relationship between muscle mass and muscle strength might not be as clear as previously thought, muscle mass is still a determinant of muscle strength [3]. Furthermore, intracellular fluid has been shown to be positively associated with muscle strength, power, and performance [66,67]. Conversely, extracellular to intracellular ratio is negatively associated with muscle strength and performance [68]. These results indicate that PhA and muscle strength are closely associated.

#### 4.1.3. Muscular Adipose Tissue

One out of the four included studies did not find an association between MAT and muscle strength. However, this non-significant finding might be due to the sample used in the study by Wroblewski et al. [32]. The sample consisted out of senior athletes (mean age: 60.1 ± 11.5 years) but there was no significant increase in IntraMAT or decrease in muscle mass commonly seen in the elderly [3,62,69,70]. In addition, despite decreased muscle strength being associated with increasing age [3,70] muscle strength did not significantly and progressively decrease when comparing the age groups in chronological order. Thus, chronic exercise prevented physiological changes associated with age in the general population and suggests that the study’s results are not applicable to the general population observed in the other included studies. Aging is commonly linked to an increase in both IntraMAT and intermuscular adipose tissue (InterMAT) [62,69] which have both been associated with a decrease in muscle strength [62,63,71,72,73]. The studies in this review did not investigate InterMAT exclusively but instead investigated IntraMAT or MAT (i.e., the sum of InterMAT and IntraMAT). Interestingly, the study by Baum et al. [33] presented similar results for IntraMAT and MAT outcomes (r range IntraMAT and muscle strength vs. MAT and muscle strength: [−0.78]–[−0.83] vs. [−0.77]–[−0.87]). Previous studies have shown possible associations between MAT and muscle strength [71,72] and these possible associations have been mechanistically linked [63,73]. Such mechanistic models indicate that a muscle’s force production is directly negatively influenced by adipose tissue accumulation within the muscle tissue [61,63,73]. Increases in MAT were found to directly result in a decrease in muscle quality (i.e., ratio of muscle strength to muscle mass [1,2]) [61,63]. It should be noted that the significance of the results of the included studies could be reduced due to a potential risk of bias within the studies. However, these results indicate that MAT is an important determinant of muscle strength.

### 4.2. Muscle Architecture

#### 4.2.1. Fascicle Pennation Angle

Three out of a total of seven studies found an association between θf and muscle strength. The remaining four studies found mixed results. For example, Selva Rai et al. [43] found a weak positive correlation between isometric knee extension MVC and θf of the quadriceps femoris muscles but not between concentric knee extension MVC and θf of the quadriceps femoris muscles. Previous studies have highlighted that changes of θf are difficult to assess from a technological perspective [74,75]. In addition, movement itself was found to change a muscle’s θf [76,77]. These factors present a significant challenge for studies and their resulting interpretations when assessing θf, especially in dynamic movements. It seems plausible that θf would be a determinant of muscle strength since θf represents the angle between muscle fascicles and the deep fascia between muscle groups [74]. Such a structure permits the accommodation of more contractile tissue in parallel within a muscle [74]. A larger θf would imply a greater number of sarcomeres in parallel and would increase the force production capacity of the muscle [74]. Increases in muscle strength and cross-sectional have been hypothesized to be explained by small increases in θf [74]. Previous studies have observed that increases in θf were associated with increases in cross-sectional area induced by resistance training [78,79,80]. While this close relationship between θf and cross-sectional area might be a factor in the possible connection between θf and muscle strength, due to the individual association between muscle mass and muscle strength [81,82,83], the inclusion of θf alongside muscle mass has also been shown to increase models predicting muscle strength [44,45]. These results indicate that θf is likely associated with muscle strength. However, technological difficulties currently hinder the reliability of assessing θf.

#### 4.2.2. Fascicle Length

Only one study out of three studies found a weak to moderate positive correlation between lf and muscle strength. However, only lf of the proximal vastus lateralis muscle correlated with both isometric and concentric knee extension MVC [44]. This finding is in line with the observation that the vastus lateralis muscle is likely the most valid individual quadricep muscle when assessing muscle recruitment in compound and isolated knee extension exercises [84]. However, the study by Trezise et al. [44] also found that lf did not improve the models for predicting muscle strength. Current literature indicates that lf is an important factor for force production during high velocity muscle contractions [85,86,87]. This is likely due to the role that lf plays in muscle fiber shortening velocity [88,89] and subsequently, the importance that the fascicle shortening velocity plays in muscular power [87,90]. Thus, greater fascicle lengths would result in greater maximal shortening velocities [86,87,91] and increase high speed performance [85,86,87]. However, the results of this study do not indicate that lf is associated with muscle strength.

#### 4.2.3. Muscle Fiber Type

Three studies assessed the correlation between muscle fiber type and muscle strength. Two studies presented mixed results, while the third study found no association between muscle fiber type and muscle strength. Interestingly, out of the two studies that did find an association between fiber type and muscle strength, one study found muscle fiber type II to be a predictor of 1RM in some groups [42], while the second study found muscle fiber type I to be correlated with isometric muscle contraction [41]. Muscle fibers are generally differentiated into three groups: type I, type IIA, and type IIX [92]. Type I muscle fibers are commonly associated with aerobic and endurance performance, whereas type II have been linked to anaerobic, power, and strength performance [93,94]. These differences have been linked to physiological differences in mitochondria volume density, length of capillary-fiber contact [95], and cross-sectional area [96,97] which ultimately result in differences in peak power [98], contractile velocity [97], and aerobic capacity [95]. In line with previous literature which associated muscle fiber type II with strength performance [93,94], Souza et al. [42] linked muscle fiber type II to muscle strength and not muscle fiber type I. However, muscle fiber type II only explained a small percentage of the variance in muscle strength. Furthermore, muscle fiber type II was only found to be a predictor of muscle strength in the low strength group and the combined group, but not in the high strength group. Previous studies have linked muscle fiber composition to athletic performance [93,99,100] but those studies predominately observed athletic populations while the studies included in this review consisted out of populations which did not participate in any systematic physical training. Maximal force production is influenced by a large range of factors [101] and it is plausible that neural factors such as muscle activation are the predominant determinants of muscle strength, especially in untrained populations [102]. In addition, untrained populations likely have an even split of muscle fiber type I and II [103] which would lessen any benefits associated with having a predominate muscle fiber type. Thus, muscle fiber type could potentially be a determinant of muscle strength, but its relevance is likely reserved for athletic populations.

### 4.3. Neuromuscular Components

#### 4.3.1. Neuromuscular Activation

Neuromuscular activation was assessed by two studies. Both studies found a moderate to strong positive correlation between muscle activation and muscle strength. Furthermore, muscle activation was found to be a predictor of muscle strength. A muscle contraction consists out of a series of events which begins with the neural excitation from the central nervous system. This then leads to the excitation-contraction coupling, which is then followed by a muscle contraction driven by the formation of cross-bridges. The result is the transmission of force through the muscle [104,105]. The studies included within this review assessed muscle activation via EMG amplitude, EMG:M and %VA. These parameters are used to assess neuromuscular excitation [106,107,108] and neglect the peripheral factors associated with neuromuscular activation which might also be associated with muscle quality [6]. For example, disturbances of calcium homeostasis negatively influence the excitation-contraction coupling [109] and other factors, such as aging, have been shown to reduce calcium sensitivity which, in turn, reduces muscle power output [110]. Thus, neuromuscular measurements instruments, such as muscle and nerve stimulation procedures [108], which assess peripheral factors are required to assess neuromuscular components relating to muscle strength.

#### 4.3.2. Motor Unit

One study investigated the relationship between muscle strength and motor unit size and number. The study did not find a main effect for either motor unit size or motor unit number on muscle strength. This result is surprising as a motor unit represents the smallest functional unit that innervates muscle fibers and controls the series of events that elicit a muscular contraction [111]. As such it plays a central role as the link between muscle and central nervous system. Motor units have been previously associated with muscle strength due to the observation that the age-related loss of muscle strength has been shown to coincide with a loss of motor units [112,113]. Interestingly, the findings of a previous study by McNeil et al. [113] indicated that the age-related loss of motor unit number might only negatively influence muscle strength at later stages of life (>80 years old). Thus, the older adult group in the included study by Kaya et al. [50] might not have been old enough (mean age older adults: 67 ± 1.20 years) for the age-related strength loss due to motor unit number or size to become a factor. This is further supported by the study’s finding that motor unit number was positively associated with muscle strength when controlling for age and gender [50]. These findings indicate that while motor unit number might influence muscle strength, its effect might only manifest when a certain amount of motor units is lost [50,113] and might not be relevant at earlier stages of life. Thus, motor units might only be relevant for muscle strength in the older population.

### 4.4. Muscle Oxidative Capacity

The relationship between muscle oxidative capacity and muscle strength was assessed by one study. The study found muscle oxidative capacity to be a significant predictor of muscle strength. Previous studies have linked mitochondrial oxidative capacity to physical function [114,115], physical activity status, and muscle quality [115]. The potential association between muscle oxidative capacity and muscle quality might be due to various factors. Mitochondria are largely responsible for the production of cellular energy and free radicals, and changes such as decreases in mitochondrial volume, mitochondrial dysfunction, and oxidative damage all reduce mitochondrial capacity [49,116]. In addition, oxidative damage likely also impairs calcium regulation, affects myofilament structure and function, and limits ATP production [49,116]. Thus, these factors either directly negatively influence a muscle’s force production ability or lower the muscle’s energy resources. Interestingly, Zane et al. [49] directly assessed muscle quality by calculating the ratio of quadriceps peak torque to thigh muscle cross-sectional area and found muscle oxidative capacity to be a predictor of muscle quality [49]. Muscle oxidative capacity might be a potential determinant of muscle strength, but further research is required to assess the association between both parameters.

### 4.5. Insulin Sensitivity

Two out of five studies found an association between IS and muscle strength. The study by Gysel et al. [39] found a weak negative correlation between muscle strength and IS, while Gysel et al. [35] found higher muscle strength outcomes in the group with higher IS. The remaining studies did not find a clear association between IS and muscle strength. The results of previous studies that assessed the relationship between IS and muscle strength were also reflective of the findings of the included studies: Some studies found a link between insulin sensitivity and muscle strength [117,118,119] and other studies did not find an association between both outcomes [37,120]. It seems plausible that insulin sensitivity would be associated with muscle strength due to the major role that muscle tissue plays in the uptake and clearance of glucose [121]. A decreased insulin sensitivity would impair insulin signaling and thus negatively affect glucose entry into muscle cells which would, in turn, decrease the synthesis of adenosine triphosphate (ATP) [122]. Thus, a lowered IS could impair ATP synthesis which would decrease a muscle’s force production capacity [123]. What has been found is that IS seems to be inversely correlated with muscle function [120] and muscle mass [37]. In addition, IS has also been shown to negatively influence muscle composition, specifically an increased amount of MAT [124,125,126] which could negatively influence muscle strength [61,63,71,72,73]. Some authors have also highlighted that lowered muscle strength might be linked to IS due to the loss of muscle strength preceding IS. Thereby, muscle weakness would lead to reductions in physical activity which would negatively impact a wide range of health-related outcomes, including IS, which would further reduce muscle strength [35,117,118]. Collectively, the results of this review indicate that IS might, if at all, have only a small direct effect on muscle strength. Rather, it seems that lowered IS is a global systemic condition that leads to multiple downstream effects which then, in turn, could negatively influence muscle strength.

### 4.6. Limitations

Limitations within this review included the following: Firstly, it is important to highlight that the included studies which investigated the relationship between a proposed factor of muscle quality and muscle strength were few in quantity and the results were largely mixed. Thus, the results of this study should be seen as tentative. Secondly, all the included studies were cross-sectional studies which limits any causal associations of the results. Thirdly, a wide range of muscle-related outcomes was covered in this review. Thus, it might be the case that some rather specific keywords, that would have helped to retrieve further relevant studies, were missed. Furthermore, although the investigated factors were previously discussed and designated as important determinants of muscle quality [6], the current work did not encompass all factors associated with muscle quality. Additionally, the search and identification of studies, as well as qualitative reviewing and summarizing of included studies were performed by only one investigator. Finally, studies which were not in the English or German language were not included within this paper. Thus, studies which investigated relevant outcomes but were published in other languages were not included and this might have negatively influenced the comprehensiveness of this paper’s results.

## 5. Conclusions

Muscle quality is commonly defined as the ratio of muscle strength to muscle mass. However, limiting the definition to relative muscle strength should be questioned, as more factors should be encompassed when talking about the relationship of muscle physiology, related physiological parameters, and functional muscle force output.

The results of the current study show that muscle composition assessed using PhA and EI is related to muscle strength and can thus be valuable indicators of muscle quality. Furthermore, MAT, θf, and muscle oxidative capacity could be promising indicators of muscle quality due to potential associations with muscle strength. These findings highlight that (i) attention should be brought to the importance of muscle quality in the healthy adult population due to the association that these muscle-associated physiological factors have on muscle strength, and (ii) muscle strength is influenced by a large array of factors which affect the force-generating capability of skeletal muscle tissue. These indicators of muscle quality should be encompassed when discussing muscle quality. Therefore, this study proposes that muscle quality be defined as all muscle-associated physiological factors that influence the force-generating capability of skeletal muscle tissue. Which specific factors should be encompassed within this definition of muscle quality will require thorough future research. Future studies should seek to establish the relationship of MAT, θf, and muscle oxidative capacity to muscle strength. When assessing these outcomes, attention should be placed on improving the reliability of θf measurements, as well as performing studies with a high methodological quality, especially when assessing MAT.

## Figures and Tables

**Table 1:** PubMed Literature Search Strategy.

Category	Search Term
Muscle Strength	(“Muscle Strength” OR Strength* OR Strong* OR “Maximum Voluntary Contraction” OR “Maximum Voluntary Isometric Contraction” OR “Torque”)
Muscle Quality	(“Muscle Quality” OR “Muscle Fib*” OR “Contractile Propert*” OR “Myosin Heavy Chain” OR “Satellite Cell” OR “Intermuscular Adipose” OR “Intramuscular Adipose” OR “Intermuscular Fat*” OR “Intramuscular Fat*” OR “Fat Infiltration” OR “Fatty Infiltration” OR “Adipose Tissue Infiltration” OR “Phase Angle” OR “Echo Intensity” OR “Muscle Density” OR “Muscle Attenuation” OR “Aerobic Capacity” OR “Insulin Resistance” OR “Insulin Sensitivity” OR “Fascicle Length” OR “Pennation Angle” OR “Motor Unit” OR “Neuromuscular Activ*” OR “EMG Amplitude” OR “Root Mean Square” OR “RMS”)

**Table 2:** Overview and Summary of the Included Studies.

Reference	Study Sample	Inclusion/Exclusion Criteria	Muscle Strength Outcome	Muscle Quality Outcome	Key Findings	Study Quality
Garrett et al., 2021 [9]	30 recreationally active college-aged young adults (f: n = 15, 19.5 ± 0.8 years, 64 ± 7.9 kg; m: n = 15, 21.1 ± 1.8 years, 83.9 ± 10.8 kg)	✕ cardiovascular, metabolic, or muscular diseases	knee EXT MVC, absolute and normalized to body mass (isometric; dynamometer)	EI, subcutaneous fat corrected (US; vastus lateralis muscle)	moderate negative correlation between EI and knee EXT MVC in the combined sample (absolute: r = −0.354, *p* = 0.028; normalized: r = −0.520, *p* = 0.002)	fair
Yamauchi et al., 2021 [10]	25 healthy young adults (f: n = 15, 21.6 ± 0.8 years, 50.2 ± 5.6 kg, 160.0 ± 5.7 cm, 19.5 ± 1.7 kg/m^2^; m: n = 10, 22.3 ± 2.4 years, 59.7 ± 3.1 kg, 171.0 ± 4.3 cm, 20.4 ± 0.8 kg/m^2^)	✓aged between 18–35 years ✕ participation in systematic training programs, walking aid, history of lower limb trauma or surgery, neuromuscular, metabolic, hormonal, or cardiovascular diseases	knee EXT MVC (concentric; dynamometer)	EI (US; rectus femoris, vastus lateralis, and vastus medialis muscles)	moderate negative correlation between knee EXT MVC and vastus medialis muscle EI (f: r = −0.63, *p* < 0.05; m: r = −0.65, *p* < 0.05)	fair
Bali et al., 2020 [11]	13 younger men (23 ± 4 years, 70.1 ± 12.1 kg, 174.1 ± 6.7 cm, 23.1 ± 3.7 kg/m^2^), 15 younger women (21 ± 2 years, 58.7 ± 9.1 kg, 162.7 ± 5.1 cm, 22.1 ± 2.8 kg/m^2^), 10 older men (73 ± 6 years, 79.2 ± 13.0 kg, 172.4 ± 3.4 cm, 26.6 ± 3.9 kg/m^2^), 15 older women (70 ± 5 years, 69.1 ± 6.3 kg, 162.5 ± 6.8 cm, 26.2 ± 2.5 kg/m^2^)	✓ aged 18–35 years and ≥65 years ✕ neurological, neuromuscular, or musculoskeletal disorders that impair the ability to perform muscle strength testing, regular lower body exercises, BMI ≥30 kg/m^2^	knee EXT MVC, absolute and normalized to cross-sectional area (concentric; dynamometer)	EI, subcutaneous fat corrected and noncorrected (US; vastus lateralis and rectus femoris muscles)	weak–moderate negative correlation between knee EXT MVC and EI (absolute knee EXT MVC and noncorrected EI: r = −0.527, *p* < 0.001; absolute knee EXT MVC and corrected EI: r = −0.453, *p* < 0.001; normalized knee EXT MVC and noncorrected EI: r = −0.335, *p* < 0.014; normalized knee EXT MVC and corrected EI: r = −0.337, *p* < 0.014) knee EXT MVC predictor of corrected EI (R = 0.453, R^2^ = 0.206, adjusted R^2^ = 0.190, *p* < 0.001)	fair
Yamaguchi et al., 2019 [12]	139 healthy community-dwelling elderly (f = 74, m = 65, median 75 years)	✓ aged ≥65 years ✕ history of conditions that affect muscle mass	HG MVC (dynamometer)	EI (US; masseter muscle)	moderate negative correlation between HG MVC and EI (r = −0.42, *p* < 0.05) HG MVC predictor of EI (β = −0.32, *p* < 0.01; R = 0.59, adjusted R^2^ = 0.3)	good
Akagi et al., 2018 [13]	20 young men (22 ± 2 years, 62.6 ± 6.5 kg, 170.6 ± 5.0 cm), 20 young women (22 ± 1 years, 51.7 ± 6.5 kg, 157.4 ± 4.1 cm), 19 elderly men (73 ± 5 years, 67.6 ± 10.3 kg, 165.4 ± 6.5 cm) and 14 elderly women (72 ± 7 years, 56.0 ± 5.9 kg, 154.5 ± 4.7 cm)	NR	plantar FLX MVC (isometric; dynamometer)	EI (US; gastrocnemius and soleus muscles)	EI predictor of plantar FLX MVC (β = −0.203, *p* = 0.036)	poor
Stock et al., 2018 [14]	23 older adults (f: n = 12, 71 ± 5 years, 26.6 ± 3.1 kg/m^2^; m: n = 11, 74 ± 7 years, 26.2 ± 3.6 kg/m^2^)	✕ metabolic or neuromuscular diseases, participation in regular resistance or aerobic training	knee EXT MVC, normalized to body mass (isometric; dynamometer)	EI, subcutaneous fat corrected and noncorrected (US; rectus femoris muscle)	moderate negative correlation between subcutaneous fat corrected EI and normalized knee EXT MVC (r = −0.5, *p* < 0.05)	fair
Gerstner et al., 2017 [15]	20 young men (20.1 ± 52 years, 71.66 ± 9.68 kg, 173.71 ± 7.47 cm) and 20 older men (69.45 ± 3.07 years, 80.77 ± 8.18 kg, 177.70 ± 6.23 cm)	✓ recreationally physically active ✕ metabolic or neuromuscular diseases, musculoskeletal injuries of the low back or lower limb	plantar FLX MVC, absolute and normalized to isometric force (concentric; dynamometer)	EI, subcutaneous fat corrected (US; gastrocnemius muscle),	moderate correlation between EI and percent decrease in plantar FLX MVC from slow to fast velocity (younger adults: r = 0.479, *p* = 0.032; older adults: r = 0.526, *p* = 0.025; groups combined: r = 0.605, *p* < 0.001)	poor
Mota & Stock, 2017 [16]	12 younger (25 ± 3 years, 65.2 ± 8.8 kg) and 13 older men (74 ± 6 years, 80.6 ± 10.4 kg)	✕ surgery on the hip or knee joints, neuromuscular or metabolic diseases, walking aids, participation in lower body resistance training or structured exercise	knee EXT MVC, absolute and normalized to body mass (isometric; tension/load cell)	EI, subcutaneous fat corrected (US; rectus femoris muscle)	moderate-strong negative correlation between EI and normalized knee EXT MVC (older men: r = −0.580, *p* = 0.038; combined sample: r = −0.733, *p* < 0.001) moderate negative correlation between EI and absolute knee EXT MVC (combined sample: r = −0.616, *p* < 0.001)	fair
Taniguchi et al., 2017 [17]	179 elderly women (74.1 ± 4.9 years, 50.0 ± 7.2 kg, 151.9 ± 5.0 cm, 21.7 ± 2.8 kg/m^2^)	✕ walking aids, history of lower limb trauma or surgery, acute disease that causes muscle weakness	knee EXT MVC (isometric; dynamometer)	EI (US; rectus femoris and vastus intermedius muscles)	weak negative correlation between knee EXT MVC and EI (r = −0.320, *p* < 0.05) EI was a predictor of knee EXT MVC (Model 1: R^2^ = 0.17, β = −0.42, standardized β = −0.17, *p* = 0.03; Model 2: R^2^ = 0.22, β = −0.38, standardized β = −0.16, *p* = 0.04)	fair
Rech et al., 2014 [18]	45 habitually physically active elderly women (70.28 ± 6.2 years, 69.02 ± 11.5 kg, 1.55 ± 0.67 cm, 27.89 ± 3.6 kg/m^2^)	✕ neurological, cardiovascular, or lower limb diseases	knee EXT MVC (isometric; dynamometer) HG MVC (dynamometer)	EI (US; rectus femoris, vastus lateralis, vastus intermedius, vastus medialis, and average quadriceps femoris muscles)	weak negative correlation between knee EXT MVC and EI (quadriceps femoris: r = −0.334, *p* < 0.05; rectus femoris: r = −0.314, *p* < 0.05; vastus lateralis: r = −0.399, *p* < 0.01; vastus intermedius: r = −0.452, *p* < 0.01; vastus medialis: r = −0.385, *p* < 0.01) weak negative correlation between MVC and HG MVC (rectus femoris: r = −0.347, *p* < 0.05)	poor
Wilhelm et al., 2014 [19]	50 healthy older men (66.1 ± 4.5 years, 1.75 ± 0.06 m, 80.2 ± 11.0 kg)	✕ metabolic and endocrine diseases, participation in any systematic physical exercise	knee EXT MVC (isometric; dynamometer) 1RM (knee extension machine)	EI (US; rectus femoris, vastus lateralis, vastus intermedius, vastus medialis, and average quadriceps femoris muscles)	moderate negative correlation between EI and 1RM (range r = [−0.498]–[−0.656], *p* ≤ 0.05), and between EI and knee EXT MVC (range r = [−0.460]–[−0.640], *p* ≤ 0.05)	fair
Watanabe et al., 2013 [20]	184 elderly men (74.4 ± 5.9 years, 62.3 ± 9.5 kg, 163.2 ± 6.0 cm)	✓ the ability to walk without assistive aid ✕ lower limb trauma or surgery, neuromuscular disorder, strength or power impairing disease	knee EXT MVC (isometric; dynamometer)	EI (US; quadriceps femoris muscles)	weak negative correlation between knee EXT MVC and EI of rectus femoris (r = −0.333, *p* < 0.001), even when controlling for age, height, weight, and subcutaneous fat thickness (r = −0.301, *p* < 0.01) weak predictive effect of EI on knee EXT (β = −0.294, *p* < 0.001)	good
Cadore et al., 2013 [21]	31 healthy elderly men (65.5 ± 5.0 years, 81.8 ± 12.0 kg, 172.2 ± 5.8 cm)	✕ participation in regular exercise training, neuromuscular, metabolic, hormonal or cardiovascular diseases	knee EXT MVC (isometric and concentric; dynamometer)	EI (US; quadriceps femoris muscles)	moderate negative correlation between EI and knee EXT MVC (isometric: r = −0.51, *p* < 0.01; concentric: r = −0.48–−0.76, *p* < 0.01)	fair
Fukumoto et al., 2012 [22]	92 elderly women (70.4 ± 6.6 years, 50.4 ± 6.2 kg, 151.1 ± 5.4 cm, 22.0 ± 2.3 kg/m^2^)	✕ walking aid, lower limb trauma or surgery, neuromuscular disorder, acute or chronic disease that impaired strength or power	knee EXT MVC (isometric; dynamometer)	EI (US; quadriceps femoris muscles)	moderate negative correlation between knee EXT MVC and EI (r = −0.40, *p* < 0.01) weak negative correlation between knee EXT MVC and EI when controlling for age and muscle thickness (r = −0.26, *p* < 0.05) weak predictive effect of EI on knee EXT MVC (R^2^ = 0.29, β = −0.73, standardized β = −0.27, *p* < 0.01)	good
Strasser et al., 2013 [23]	52 lower-limb healthy younger (24.2 ± 3.7 years, 70.2 ± 15.1 kg, 1.8 ± 0.1 m) and older adults (67.8 ± 4.8 years, 77.2 ± 13.2 kg, 1.7 ± 0.1 m)	✕ neuromuscular diseases, prosthesis or fractures of the lower extremities, injuries or pain of the lower limb	knee EXT MVC (isometric; load cell)	EI (US; rectus femoris, vastus lateralis, vastus intermedius, and vastus medialis muscles) θf (see above)	moderate positive correlation between knee EXT MVC and vastus intermedius muscle EI in younger adults (r = 0.68, *p* < 0.001) moderate negative correlation between knee EXT MVC and θf in younger adults (r = [−0.47]–[−0.64], *p* ≤ 0.05) positive interaction between knee EXT MVC and θf vastus intermedius muscle (*p* ≤ 0.01)	good
Kolodziej et al., 2021 [24]	346 elderly adults (f: n = 259, 64.3 ± 5.8 years, 70.4 ± 12.2 kg, 159.5 ± 5.8 cm, 27.7 ± 4.6 kg/m^2^; m: n = 87, 66.3 ± 6.9 years, 85.6 ± 13.7 kg, 174.0 ± 7.0 cm, 28.2 ± 3.8 kg/m^2^)	✓ aged ≥50 years ✕ medical contraindication, difficulty walking or limitations in daily activities, BMI ≥50 kg/m^2^, metal prostheses or limb amputations	knee EXT MVC (isometric; dynamometer) HG MVC, normalized to appendicular muscle mass (dynamometer)	PhA (BIA)	older participants had lower PhA and strength values when compared to younger participants (*p* < 0.001) PhA had a weak predictive effect on HG MVC (R^2^ = 0.692, β = 0.095, *p* = 0.040) and knee EXT MVC (R^2^ = 0.452, β = 0.132, *p* = 0.034)	fair
Matias et al., 2021 [25]	94 overweight, former top-level athletes (f: n = 32, 43.5 ± 8.7 years, 81.7 ± 12.2 kg, 163.0 ± 6.3 cm, 30.7 ± 3.9 kg/m^2^; m: n = 62, 42.8 ± 9.8 years, 98.2 ± 17.9 kg, 175.9 ± 6.7 cm, 31.7 ± 5.1 kg/m^2^)	✓ BMI ≥ 25 kg/m^2^, physically inactive ✕ cardiovascular or psychological disorders	knee EXT MVC (isometric; leg press) HG MVC (dynamometer)	PhA (BIA)	moderate positive correlation between PhA and HG MVC (r = 0.556, *p* < 0.001) and knee EXT MVC (r = 0.422, *p* < 0.001) PhA had a predictive effect on HG MVC (R^2^ = 0.708, β = 2.846, *p* = 0.012) and knee EXT MVC (R^2^ = 0.275, β = 24.209, *p* = 0.041)	good
Bittencourt et al., 2020 [26]	152 community-dwelling older women (71 ± 4.38 years, 69.4 ± 12.01 kg, 1.56 ± 0.07 m, 28.4± 4.25 kg/m^2^)	NR	HG MVC (dynamometer)	PhA (BIA)	weak positive correlation between PhA and HG MVC (r = 0.177, *p* = 0.029)	poor
Di Vincenzo et al., 2020 [27]	12 female volleyball players (23.8 ± 3.6 years, 63.0 ± 5.1 kg, 170 ± 4 cm, 21.9 ± 1.3 kg/m^2^) and 22 non-athletic females (23.6 ± 2.0 years, 60.7 ± 4.8 kg; 167 ± 5 cm; 21.9 ± 1.3 kg/m^2^))	NR	HG MVC (dynamometer)	PhA (BIA, upper limbs and whole body)	moderate positive correlation between HG MVC and whole body PhA (r = 0.696, *p* = 0.012) and upper limb PhA (r = 0.821, *p* = 0.001) in all subjects	poor
Hetherington-Rauth et al., 2020 [28]	249 adults (f: n = 158, 42.4 ± 11.5 years, 24.0 ± 4.1 kg/m^2^; m: n = 91, 41.1 ± 13.0 years, 25.6 ± 3.8 kg/m^2^) and 75 older adults (f: n = 54, 75.7 ± 7.8 years, 28.6 ± 4.3 kg/m^2^; m: n = 21, 75.7 ± 7.3 years, 28.8 ± 3.3 kg/m^2^)	✕ health problems that contraindicate muscle performance tests, mobility limitations	HG MVC (dynamometer)	PhA (BIA)	no association between PhA and HG MVC in both adult groups	fair
Bourgeois et al., 2019 [29]	146 adults (f: n = 86, 49 ± 16 years, 72.9 ± 17.6 kg, 162.8 ± 6.8 cm, 27.6 ± 6.9 kg/m^2^, m: n = 60, 45 ± 18 years, 87.3 ± 17.0 kg, 176.9 ± 6.9 cm, 27.9 ± 5.2 kg/m^2^)	✓ aged ≥18 years ✕ no medical implants, joint replacements, underlying chronic diseases, body weight ≥200 kg	knee EXT MVC (concentric; dynamometer) HG MVC (dynamometer)	PhA (BIA)	PhA was a predictor of HG MVC (right: R^2^ = 0.66, β = 2.93, *p* < 0.01; left: R^2^ = 0.61, β = 2.62, *p* < 0.01) and knee EXT MVC (right leg: R^2^ = 0.71, β = 11.12, *p* < 0.0001)	fair
Rodrígues-Rodrígeuz et al., 2016 [30]	223 healthy, non-athlete adult men (27 ± 10 years, 65.0 ± 11.3 kg, 1.68 ± 0.08 m, 22.8 ± 2.9 kg/m^2^)	✕ inflammatory joint disease, neurological disorder, injury of the upper extremities, major systematic disease, elite level athletic participation	HG MVC, absolute and normalized to bodyweight (dynamometer)	PhA (BIA)	moderate positive correlation between PhA and HG MVC (absolute: r = 0.582, *p* < 0.05; normalized: r = 0.425, *p* < 0.05)	fair
Young et al., 2016 [31]	42 adults (f = 26, m = 16, 24.9 ± 11.4 years, 23.3 ± 3.0 kg/m^2^)	✓ varying activity levels ✕ medical conditions which would make participation unsafe	knee EXT and FLX MVC, absolute and normalized to body weight (isometric; dynamometer)	IntraMAT (EI/US; rectus femoris and biceps femoris muscles)	moderate negative correlation between rectus femoris IntraMAT and normalized knee EXT MVC (r = −0.4, *p* = 0.01) moderate negative correlation between bicep femoris IntraMAT and knee FLX MVC (absolute: r = −0.4, *p* = 0.01; normalized: r = −0.5, *p* < 0.01)	poor
Wroblewski et al., 2011 [32]	40 competitive masters athletes (40–49: f = 5, m = 5, 45.9 ± 3.1 years, 136.3 ± 18.1 lbs, 20.3 ± 1.3 kg/m^2^; 50–59: f = 5, m = 5, 54.4 ± 3.5 years, 144.2 ± 25.2 lbs, 21.9 ± 2.8 kg/m^2^; 60–69: f = 5, m = 5, 65.2 ± 2.5 years, 134.8 ± 21.7 lbs, 21.6 ± 2.2 kg/m^2^; 70 +: f = 5, m = 5, 75.4 ± 3.4 years, 135.7 ± 19.18 lbs, 22.9 ± 1.5 kg/m^2^)	NR	knee FLX MVC (isometric; dynamometer)	IntraMAT (MRI; quadriceps femoris muscles)	no correlation between MVC and IntraMAT	fair
Baum et al., 2016 [33]	9 adult men (28 ± 8 years, 28.1 ± 3.9 kg/m^2^)	✕ diabetes, neuromuscular disorders or quadriceps muscle injuries	knee EXT MVC, at 60° and 90° knee FLX (isometric; dynamometer)	IntraMAT (MRI; quadriceps femoris muscles) MAT (PDFF/MRI; quadriceps)	strong negative correlation between knee EXT MVC and IntraMAT (60°: r = −0.78, *p* = 0.013; 90°: r = −0.83, *p* = 0.006) strong negative correlation between knee EXT MVC and PDFF (60°: r = −0.77, *p* = 0.015; 90°: r = −0.87, *p* = 0.002)	poor
Inhuber et al., 2019 [34]	20 moderately active, healthy adults (f = 10, m = 10; age range: 22–41 years; BMI range: 22.2–31.8 kg/m^2^)	✓ aged between 20–45 years, BMI between 23–33 kg/m^2^ ✕ history of high-performance sports, or neuromuscular or metabolic diseases, previous knee or thigh injuries	knee EXT and FLX MVC, normalized to BMI (isometric; dynamometer)	MAT (PDFF/MRI; bilateral thigh muscles)	moderate negative correlation between knee MVC and PDFF (range r = [−0.446]–[−0.676], *p* < 0.05) PDFF predictor of knee EXT and FLEX MVC bilaterally (*p* < 0.05)	poor
Gysel et al., 2014 [35]	178 healthy adult men (more insulin sensitive: n = 89, 33.2 + −5.4 years, 76.0 ± 8.18 kg, 1.80 ± 6.18 m, 23.4 ± 3.3 kg/m^2^; less insulin sensitive: n = 89, 35.6 + −5.3 years, 91.0 ± 13.7 kg, 1.79 ± 6.57 m, 28.2 ± 3.9 kg/m^2^)	✕ illnesses or medication that may affect body composition, bone metabolism or sex steroid levels	knee EXT and FLX MVC, absolute and normalized to muscle mass (concentric; dynamometer) elbow EXT MVC and elbow FLX MVC, absolute and normalized to muscle mass (concentric; dynamometer) HG MVC, normalized to muscle mass (dynamometer)	IS (HOMA-IR)	greater absolute elbow EXT MVC for the less insulin sensitive (+8%, *p* < 0.05) normalized HG MVC, knee EXT MVC and knee FLX MVC, and elbow EXT MVC and FLX MVC were lower in the less insulin sensitive (*p* < 0.05)	good
Justice et al., 2014 [36]	56 elderly adults (f: n = 34, 75.8 ± 6.0 years, 26.3 ± 4.9 kg/m^2^; m: n = 22, 74.7 ± 6.1 years, 27.3 ± 2.7 kg/m^2^)	✕ diabetes, neurological disorders, chronic pain, advanced chronic diseases, medical condition which would limit safe participation or BMI >40 kg/m^2^	knee EXT MVC and FLX MVC, normalized to body weight (isometric) dorsal EXT MVC, normalized to body weight (isometric) 1RM dorsal EXT, normalized to body weight	IS (Minimal Model Identification)	weak positive correlation between IS and 1RM (r = 0.30, *p* < 0.05)	good
Bijlsma et al., 2013 [37]	301 low to highly active, healthy, elderly adults (f: n = 155, 64.4 ± 7.7 years, 71.9 ± 11.2 kg, 1.66 ± 0.06 m, 26.0 ± 4.1 kg/m^2^; m: n = 146, 67.4 ± 7.1 years, 83.9 ± 11.2 kg, 1.78 ± 0.06 m, 26.4 ± 3.3 kg/m^2^)	✕ neurologic disorders, metabolic diseases, rheumatic diseases, malignancy, heart failure, severe chronic obstructive pulmonary disease or recent orthopedic surgery	HG MVC (dynamometer)	IS (HOMA-IR)	no association between HG MVC and HOMA-IR	fair
Seko et al., 2019 [38]	elderly adults (f: n = 156, 74.9 ± 6.8 years, 50.8 ± 8.5 kg, 149.5 ± 5.9 cm, 22.7 ± 3.2 kg/m^2^; m: n = 116, 75.0 ± 6.4 years, 62.8 ± 11.0 kg, 163.6 ± 6.2 cm, 23.4 ± 3.6 kg/m^2^)	✓ aged ≥65 years ✕ diabetes type 2	HG MVC (dynamometer) knee EXT MVC (isometric; dynamometer)	IS (HOMA-IR)	no correlation between either HG MVC or knee EXT MVC and HOMA-IR	good
Gysel et al., 2016 [39]	178 healthy men (more insulin sensitive: 33.2 ± 5.4 years, 76.0 ± 8.27 kg, 1.80 ± 0.063 m, 23.4 ± 2.3 kg/m^2^; less insulin sensitive: 35.5 ± 5.3 years, 90.4 ± 12.56 kg, 1.79 ± 0.064 m, 28.1 ± 3.7 kg/m^2^)	✕ diseases or medication that affect body composition, bone metabolism or sex steroid levels	HG MVC, absolute and normalized to muscle mass (dynamometer)	IS (HOMA-IR)	weak negative correlation between HOMA-IR and normalized HG MVC (r = −0.23, *p* < 0.001)	fair
Herda et al., 2019 [40]	22 healthy individuals (20.4 ± 2.1 years, 172.3 ± 10.3 cm; 70.8 ± 17.0 kg)	✕ participation in structured exercise in the previous	knee EXT MVC (isometric and concentric; dynamometer)	MHC isoform (types I, IIA, and IIX; vastus lateralis muscle biopsy) MUAP (vastus lateralis EMG)	strong positive correlation between knee EXT MVC and MUAP (isometric: r = 0.81; concentric: r = 0.79, both *p* < 0.001) moderate correlation between isometric knee EXT MVC and type 1%MHC (r = 0.54, *p* = 0.048)	poor
Evangelidis et al., 2017 [41]	31 low to moderately active adults (21 ± 3 years, 1.79 ± 0.07 m, 71.8 ± 7.3 kg)	✕history of musculoskeletal problems or injuries of the lower back and lower limb	knee FLX MVC (isometric; dynamometer)	MHC isoform (muscle biopsy; biceps femoris muscle)	no correlation between MHC composition and knee FLX MVC	fair
de Souza et al., 2012 [42]	50 physically active, male, college students (23.9 ± 5.2 years, 73.2 ± 13.2 kg, 174.1 ± 6.3 cm)	✕ participation in regular strength or endurance training, health problems or neuromuscular disorders	1RM (isometric; leg press)	muscle fiber quantification (muscle biopsy; vastus lateralis muscle)	percentage of type II fibers and total muscle cross-sectional area were significantly associated with predicting muscle strength in low strength performance group (adjusted R^2^ = 0.25, *p* = 0.002) and the whole sample (adjusted R^2^ = 0.35, *p* = 0.0001)	good
Selva Raj et al., 2017 [43]	36 elderly adults (f: n = 16, 68.0 ± 5.9 years, 161.1 ± 5.9 cm, 68.9 ± 9.5 kg, 26.6 ± 3.4 kg/m^2^; m: n = 20, 68.4 ± 4.9 years, 171.6 ± 9.6 cm, 81.4 ± 12.6 kg, 27.6 ± 3.1 kg/m^2^)	✕ relevant cardiovascular or orthopedic problems, performance influencing medication or walking aids	knee EXT MVC (isometric and concentric; dynamometer)	θf (US; vastus lateralis muscle) lf (see above)	weak positive correlation between isometric knee EXT MVC and θf (r = 0.36, *p* < 0.05)	fair
Trezise et al., 2016 [44]	56 healthy men (29.0 ± 5.1 years, 1.78 ± 0.06 m, 78.6 ± 14.0 kg) consisting out of 14 runners, 13 weightlifters, 15 recreationally active, and 14 untrained	✕ cardiovascular and inflammatory diseases, lower limb injury, and performance-influencing conditions	knee EXT MVC (isometric and concentric; dynamometer/load cell)	θf [US; rectus femoris, vastus lateralis (proximal, mid, and distal), vastus intermedius muscle] EMG:M (rectus femoris, vastus lateralis, vastus medialis, and average quadriceps femoris muscles) lf (see above) %VA (interpolated twitch method of quadriceps femoris muscles activation)	weak positive correlation between θf and isometric MVC (range: r = 0.31–0.39, *p* ≤ 0.05), and isokinetic MVC (r = 0.28–43, *p* ≤ 0.05) weak–moderate positive correlation between lf and isometric MVC (r = 0.31–0.46, *p* ≤ 0.05), and concentric MVC (r = 0.31–0.37, *p* ≤ 0.05) weak–moderate positive correlation between MA and isometric MVC (range: r = 0.35–0.47, *p* ≤ 0.01), and MA and concentric MVC (range: r = 0.25–0.30, *p* ≤ 0.05) weak correlation between knee EXT MVC and %VA (isometric: r = 0.25; concentric: r = 0.27, *p* ≤ 0.05) θf vastus lateralis mid, MA average and %VA were included in the best fit model for predicting isometric MVC (R^2^ = 0.72) θf vastus lateralis proximal predictor of concentric MVC (R^2^ = 0.65)	fair
Ando et al., 2015 [45]	11 healthy men (21.9 ± 0.9 years, 174.3 ± 6.2 cm, 65.1 ± 9.3 kg)	✕ involvement in resistance training	knee EXT MVC (isometric; dynamometer)	θf (US; rectus femoris, vastus lateralis, vastus intermedius, and vastus medialis muscles) lf (see above)	moderate positive correlation between knee EXT MVC and lateral vastus intermedius θf (r = 0.68, *p* < 0.05)	poor
Cuesta-Vargas & González-Sánchez, 2014 [46]	46 healthy adult participants (f: n = 25, 30.39 ± 7.4 years, 57.9 ± 6.7 kg, 165.8 ± 5.2 cm, 24.84 ± 2.87 kg/m^2^; m: n = 21, 30.39 ± 8.2 years, 78.6 ± 14.4 kg, 178.1 ± 6.7 cm, 21.61 ± 3.44 kg/m^2^)	✕ spinal disorders, infections, osteoporotic fractures, neoplastic, metastatic or arthritic diseases, and BMI >35 kg/m^2^	lumbar EXT MVC (concentric; dynamometer)	θf (US; erector spinae muscles)	moderate predictive effect of left θf on lumbar EXT MVC (R^2^ = 0.680, standardized β = 0.443, *p* = 0.025)	good
Wakahara et al., 2013 [47]	22 healthy young men (26.0 ± 3.7 years, 68.9 ± 9.5 kg, 172.5 ± 5.1 cm)	✕ participation in regular upper extremity resistance training for at least 1 year	elbow EXT MVC, absolute and normalized to muscle mass (isometric; dynamometer/load cell)	θf (US; triceps brachii muscle)	moderate positive correlation between θf and absolute elbow EXT MVC (r = 0.471, *p* < 0.05)	poor
Cuesta-Vargas & González-Sánchez, 2013 [48]	46 healthy adults (f = 25, m = 21, 30.39 ± 7.79 years, 73.59 ± 21.20 kg, 170.52 ± 16.93 m, 23.71 ± 3.16 kg/m^2^)	NR	lumbar EXT MVC, at light, moderate, and maximal intensity (isometric; load cell)	θf (US; erector spinae muscles) MA (EMG; erector spinae muscles)	moderate-strong positive correlation between lumbar EXT MVC and θf (range: r = 0.858–0.907, *p* ≤ 0.01) moderate-strong positive correlation between lumbar EXT MVC and MA (r = 0.726–0.852, *p* ≤ 0.01) at the corresponding intensities	good
Zane et al., 2017 [49]	326 adults (f = 172, m = 154; 71.4 ± 12.6 years)	✕ major chronic conditions or functional impairments	knee EXT MVC (isometric; dynamometer)	mitochondrial oxidative capacity, via phosphocreatine resynthesis rate (phosphorus magnetic resonance spectroscopy; vastus lateralis muscle)	kPCr(*p* = 0.036) and %PCr depletion (*p* < 0.001) were greater in the higher muscle strength tertiles kPCr predictor of knee EXT MVC (adjusted R^2^ = 0.531, β = 0.114, *p* = 0.007)	fair
Kaya et al., 2013 [50]	18 older adults (f = 12, m = 6; 67 ± 1.20 years, 69.7 ± 2.77 kg, 167.7 ± 1.98 cm) and 24 younger adults (f = 10, m = 14, 22 ± 0.74 years, 72.6 ± 2.39 kg, 173.2 ± 2.30 cm)	✕ neurological or orthopedic conditions and participation of resistance training	pinch-grip MVC (isometric; force transducer)	motor unit number index (MUNIX) (SEMG; palmar hand) motor unit size Index (MUSIX) (see above)	no main effect of MUNIX or MUSIX on pinch-grip MVC	fair

Summary of all included studies with the studies’ inclusion (✓) and exclusion (✕) criteria, the most relevant findings, the investigated outcomes, and the methodological study quality, sorted by outcome parameters. %PCr, percentage phosphocreatine; 1RM, one-repetition maximum; BIA, bioimpedance analysis; EI, echo intensity; EMG:M, EMG amplitude normalized to M-wave amplitude; EXT, extension; f, female; FLX, flexion; HG, hand grip; HOMA-IR, Homeostatic Model Assessment for Insulin Resistance; IntraMAT, intramuscular adipose tissue; IS, insulin sensitivity; kPCr, postexercise phosphocreatine resynthesis rate; lf, fascicle length; m, male; MA, muscle activation; MAT; muscular adipose tissue; MHC, myosin heavy chain; MUAP, motor unit action potential; MVC, maximum voluntary contraction; NR, not reported; PDFF, proton; density fat fraction; PhA, phase angle; SEMG, surface EMG; US, ultrasound; VA, voluntary activation; years, years; θf, fascicle pennation angle. The study results were grouped into five sections in accordance with the muscle quality factor that the individual studies investigated: (I) Muscle Composition (i.e., echo intensity (EI), phase angle (PhA), muscular adipose tissue (MAT), (II) Muscle Architecture (i.e., fascicle pennation angle (θf), fascicle length (lf), muscle fiber type), (III) Muscle Oxidative Capacity, (IV) Insulin Sensitivity (IS), and (V) Neuromuscular Components (i.e., neuromuscular activation and motor unit).

## Data Availability

Not applicable.
